# Melamine Disrupts Acetylcholine-Mediated Neural Information Flow in the Hippocampal CA3–CA1 Pathway

**DOI:** 10.3389/fnbeh.2021.594907

**Published:** 2021-02-18

**Authors:** Wei Sun, Peidong Liu, Chunzhi Tang, Lei An

**Affiliations:** ^1^Medical College of Acupuncture-Moxibustion and Rehabilitation, Guangzhou University of Chinese Medicine, Guangzhou, China; ^2^Behavioral Neuroscience Laboratory, The First Affiliated Hospital of Guizhou University of Traditional Chinese Medicine, Guiyang, China; ^3^Department of Neurology, The First Affiliated Hospital of Guizhou University of Traditional Chinese Medicine, Guiyang, China

**Keywords:** acetylcholine, hippocampus, local field potential, melamine, neural information flow

## Abstract

Considering the cognitive and synaptic deficits following intragastric administration of melamine, the aim of the current investigation was to test whether the hippocampal oscillations were affected. The local field potential (LFP) was recorded in the hippocampal CA3–CA1 pathway of Wistar rats during a spatial-dependent Y-maze task. The general partial directed coherence (gPDC) method was used to assess the directionality of neural information flow (NIF) between the CA3 and CA1 regions. The levels of acetylcholine (ACh) and its esterolytic protease, acetylcholinesterase (AChE), were detected in the hippocampus (HPC) following the behavioral test. The values of phase synchronization between the CA3 and CA1 regions in delta, low theta, and high theta oscillations were reduced significantly in the melamine-treated group. Moreover, the coupling directional index and the strength of CA3 driving CA1 were critically decreased in the above three frequency bands as well. Meanwhile, a reduction in ACh expression and an enhancement in AChE activity were found in the HPC of melamine-treated rats. Intrahippocampal infusion with ACh could mitigate the weakened neural coupling and directional NIF in parallel with spatial learning improvements. However, infusion of scopolamine, an acetylcholine receptor antagonist, could block the mitigative effects of ACh treatment in melamine rats. These findings provide first evidence that ACh-mediated neuronal coupling and NIF in the CA3–CA1 pathway are involved in spatial learning deficits induced by chronic melamine exposure.

## Introduction

Melamine is a triazine heterocyclic chemical and has been widely used in the manufacturing of plastics. It cannot be used as an additive, although melamine is approved as a food-content substance. Melamine attracted wide public concern when melamine-contaminated milk products induced nephrotoxicity in animals and infants who had consumed them (Parry, 2008; Skinner et al., 2010). Refuting previous views that melamine toxicity is limited to renal function, several evidences suggest that it can disturb the functioning of the central nervous system and induce cognitive impairments (Kim et al., 2011; Yang et al., 2012; Chu et al., 2013; Bolden et al., 2017). Melamine can distribute to multiple brain regions including the cerebellum, striatum cortex, and the hippocampus (HPC) by passing through the blood–brain barrier (BBB) (Wu et al., 2009). Among the various brain regions, HPC is the most affected (An et al., 2012a; An et al., 2015). For instance, melamine induced pathological changes in HPC neurons such as the dense chromatin and shrinkage occurred mainly due to the formation of insoluble metabolites in cells (Wang et al., 2011; An and Zhang, 2014b, 2016; An et al., 2014). Pubescent rats showed learning disabilities and deficits in memory formation following long-term melamine treatment (An et al., 2011; An and Sun, 2017a). Further studies implied that melamine contamination during the prenatal period could inhibit the activation of postsynaptic receptors, thereby affecting hippocampal synaptic plasticity in the CA3–CA1 pathway, leading to cognitive deficits (An and Zhang, 2014a; An and Sun, 2017b, c). However, the underlying molecular mechanism of the destructive effects of melamine on the CA3–CA1 pathway is still unknown.

Correlated and synchronous activities in populations of neurons have been shown to play a critical role in cortical coding, network dynamics, and cognitive processing (Vogels et al., 2005; Canolty and Knight, 2010). Specifically, neurons in the hippocampus show different kinds of oscillation, and each of them with different dominant frequencies (Buhl et al., 2003; Lubenov and Siapas, 2009). Rhythms in the delta, theta, and gamma bands play significant roles in spatial and contextual memory (Tiesinga et al., 2001; Trimper et al., 2014; Chaieb et al., 2015). For example, delta frequency is associated with decision-making behavior (Basar et al., 2001). Increased theta power correlates with successful memory retrieval, while high theta oscillation occurs during animal active exploration and memory encoding (Korotkova et al., 2018; Vivekananda et al., 2020). Gamma rhythm is a high-frequency oscillation and is critical for memory retrieval and planning (Pfeiffer and Foster, 2013). Cortical synchronous activity can be generated intrinsically and regulated by neuromodulators that can cause switching between oscillatory frequencies (Salinas and Sejnowski, 2001; Averbeck and Lee, 2004). Such changes in correlated activity might reflect changes in the functional connectivity of a circuit (Salinas and Sejnowski, 2001). Interestingly, the cholinergic tone could modulate ongoing hippocampal activities by enhancing the excitatory and depressing inhibitory transmissions in the HPC, thus increasing the excitatory output to promote theta generation in HPC circuitry (Young and Jackson, 2011; Gu et al., 2017). Previous findings revealed that chronic melamine treatment in young rats could induce impairments of cognitive flexibility, including reversal learning and memory (An et al., 2013b). The damage caused by melamine to hippocampal function occurs through enhanced acetylcholinesterase (AChE) activity and perturbed acetylcholinergic transmission in the CA3–CA1 pathway (An et al., 2013b). Given that modulating the level of acetylcholine (ACh) can cause changes in synchronous activity, which were correlated with distinct cognitive functions, such as learning ability, attention, and memory consolidation (Picciotto et al., 2012; Sparks and Chapman, 2013; Teles-Grilo Ruivo and Mellor, 2013; Ma et al., 2020), it may be plausible that melamine influences neuronal activity in the hippocampus. Little is known about whether neural coupling or the neural information flow (NIF) was affected.

Here, we found that coupling in the HPC, and the coupling directional index from CA3 to CA1 of melamine-treated rats, was significantly declined in low-frequency bands, including delta, low theta, and high theta. Furthermore, we found that melamine reduced the expression of ACh in the hippocampus involved in the weakened NIF, as indicated by the intrahippocampal infusion of ACh that could effectively enhance the diminished phase synchronization and directional index of NIF induced by melamine treatment, leading to the improvement of spatial learning performance in the Y-maze task. Meanwhile, scopolamine, an acetylcholine receptor antagonist, could effectively block the mitigative effects of ACh treatment. Thus, our findings provide a new insight into the neurotoxic mechanism by which melamine affects cognitive function.

## Experimental Procedure

### Experimental Animals

Male Sprague–Dawley rats, 3 weeks old, were purchased from the Laboratory Animal Center, Academy of Military Medical Science of People’s Liberation Army, and reared in plastic cages in a colony room (21 ± 2°C, 45 ± 5% humidity, lights on at 7:00 a.m.) with *ad libitum* access to water. Except for several days before and during the behavioral test, rats were food restricted to maintain 85% free-feeding weight. All experiments were performed during the light period (between 1400 and 1700 h). Animal care and experimental procedures were approved by the Care and Use of Animals Committee of Guizhou University of Chinese Medicine (SCXK-2013-0020). The biochemical and behavioral (recording) experiments were conducted when rats were around 8 or 9 weeks old.

**FIGURE 1:**
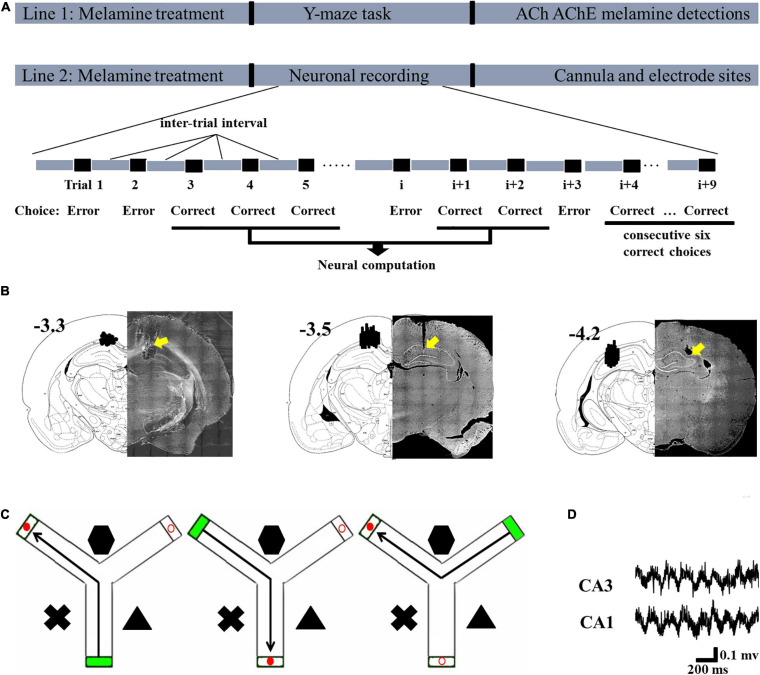
Schematic describing the experimental workflow and schematic representations of the cannula and electrode sites. **(A)** In Line 1, following modeling, the rats in each group were tested in the Y-maze task and their hippocampi were collected to assess the levels of acetylcholine (ACh), acetylcholinesterase (AChE), and melamine. In Line 2, following modeling, neuronal recording was conducted. The signal during the correct choices, excluding the inter-trial interval, was selected for statistical analysis. Since this study was focused on the learning process and not the performance after sufficient training, the signal during the last six correct trials was not included in the statistical analysis. After the recording, the placements of the cannulae and electrodes were verified. **(B)** Schematic representation of the cannula placements (*left*, –3.3) and electrode placements presented for CA1 (*middle*, –3.5) and CA3 (*right*, –4.2). **(C)** Schematic illustrations of the correct choice in the Y-maze task. *Green box* indicates the start arm; *red solid circular* indicates the correct (reward) arm; *red hollow circular* indicates the incorrect arm. **(D)** Representative local field potential (LFP) of the CA1 and CA3 regions during the correct choice.

Rats were randomly divided into five groups: control [*n* = 8 for biochemical tests and behavioral task, *n* = 5 for local field potential (LFP) recording]; melamine (*n* = 8 for biochemical tests and behavioral task, *n* = 3 for LFP recording); melamine+ACh (*n* = 8 for behavioral task, *n* = 4 for LFP recording); melamine(ACh)+SC(scopolamine) (*n* = 6 for behavioral task, *n* = 5 for LFP recording); and control cage (*n* = 4 for behavioral task, *n* = 3 for LFP recording). The rats in the melamine group were intragastrically administered with melamine solution [30 mg/ml, dissolved in 1% carboxymethylcellulose (CMC); Hongjin Chemical, Qingdao, China] at a dose of 300 mg kg^–1^ day^–1^ for four consecutive weeks, while the rats in the control group received the same dose of 1% CMC. A total of 30 min before the behavioral test, acetylcholine chloride (5.0 nM; Sigma-Aldrich, MO, United States) was bilaterally infused into the HPC of rats (0.25 μl/side) in the melamine+ACh group. A total of 15 min following acetylcholine chloride treatment, scopolamine hydrobromide (25 μg/0.25 μl; Sigma Chemicals, United States) was injected into the HPC of rats (0.25 μl/side) in the melamine(ACh)+SC group. In the bilateral infusion experiments, artificial cerebral spinal fluid (ACSF) was used as the vehicle for the acetylcholine chloride infusion in the control group and was also used as the vehicle for the scopolamine infusion in the melamine+ACh group. The doses of acetylcholine chloride and scopolamine hydrobromide were determined from published studies (Wallenstein and Vago, 2001; De Bundel et al., 2008; An et al., 2011, 2013b; Wang et al., 2015) and pilot experiments conducted in our laboratory (Supplementary Figure 1). Rats in the control cage group received no drug treatment, but left in their home cage as the control environment during the neuronal recording test. The schematic of the workflow is presented in Figure 1A.

### Spatial Learning in the Y-Maze Task

One day after the last treatment, the rats were trained in the Y-maze task (Figure 1). Three black Plexiglas arms (40 cm × 15 cm × 8 cm) separated by 120°; angles were built as the maze. Two of these were the test arms and one was the start arm. Various distal spatial cues were decorated in the experimental room. An attached start box built of black Plexiglas (18 cm × 14 cm × 14 cm) was separated from each entry arm by a removable blocker. One black bowl (4.5 cm in height, 9 cm in diameter) within the reward (0.5 g of chocolate chips; Milka, Kraft Foods) was placed 1 cm before the end of the test arm. A blocker located halfway down each arm could be operated manually from the experimenter’s position and was used to allow animals only one choice in each training trial.

The procedure was conducted as in our previous studies, with modifications (An et al., 2018; Sun et al., 2018b, 2020). Briefly, the rat was placed in the start arm and allowed to visit the end of the reward arm. For each individual animal, the reward location was fixed; however, the start arm and the test arms were pseudo-randomly selected but counterbalanced across rats of groups. After reaching the end of an arm, the rat was returned to its home cage that served as the inter-trial box. The arms were cleaned with 70% alcohol following each trial. A visit was defined as the animal placing all four paws. The inter-trial interval was about 20 s. On one single learning day, the rats were trained till they got six correct trials in a row. The total trials to reach the criterion, total time spent in reaching the criterion, and the velocity to reach the reward cup were quantified. One week after the training, a probe session (10 trials) was conducted to test the spatial memory of subgroups of rats. The percentage of the correct trials was calculated.

### Quantitation of Melamine in the Hippocampus

After the behavioral experiments, the rats in the melamine group were deeply anesthetized and the brains were removed individually. Subsequently, the HPC on the side of record was isolated on an ice-cold operation table. After being weighed, it was rinsed in 0.1 M phosphate buffer (pH 7.4) and homogenized with ice-cold saline to be 10% (*w*/*v*) homogenates. The mixtures were homogenized using a glass homogenizer for 5 min and centrifuged at 3,000 rpm at 4°;C for 15 min. The supernatant was collected and stored at −70°;C. The detections of melamine, ACh, and AChE used the same homogenates of hippocampal samples which were collected following the behavioral task.

As in a previous method (An et al., 2011), the level of melamine was determined by using an ELISA kit according to the instructions provided by the manufacturer (cat. no. sc0067/sc0068, Huaan Magnech Bio-Tech Co., Ltd., Beijing, China). Briefly, 50 μl detector antibody and 50 μl horseradish peroxidase (HRP) conjugate were added into 50 μl samples. After incubating for 30 min, we discarded the liquid by rigorously flicking and bolting the remaining liquid. Detection reagents A and B were added and the liquid was incubated for 15 min in the dark. Finally, we read the optical density absorbance at 450 nm within 3 min of adding the stop solution. The detection limit of the kit was 10 ppb. Data were expressed as micrograms per gram of hippocampus.

### ACh and AChE Content in the Hippocampus

After the behavioral experiments, the levels of ACh (cat. no. A105-1, Jiancheng Bioengineering Institute, Nanjing, China) and AChE (KL-E17521m, Kanglang Biotechnology Co., Ltd., Shanghai, China) were determined according to the methods described in the references by using commercial ELISA kits. Briefly, the samples were first diluted with a coating buffer (10 mM phosphate, pH 7.8, containing 144 mM NaCl and 0.02% NaN_3_). Wells were coated with 100 μl of the diluted specimens at 4°;C for 24 h and then rinsed twice with a washing buffer (10 mM sodium phosphate, pH 7.2, containing 0.05% Tween-20). Post-coating was carried out at 37°;C for 60 min by the addition of 200 μl/well of the coating buffer containing 1% bovine serum albumin, washing three times, and 100 μl/well of mAb diluted in the washing buffer was added and incubated at 37°;C for 60 min. Following washing three times, 100 μl/well of the second antibody solution was added to the plate and incubated at 37°;C for 60 min. After washing three times, 100 μl/well of a solution of 0.1% *o*-phenylenediamine dihydrochloride containing 0.03% H_2_O_2_ in substrate buffer was added to the wells. The reaction was allowed to proceed at room temperature for 15 min. Optical density was read at 490 nm. The protein levels of the samples were measured by the Coomassie Brilliant Blue G-250 method with bovine serum albumin as the standard.

### Bilateral Microinjection

Rats were anesthetized with isoflurane and prepared for surgery as previously described (An et al., 2012b; An and Zhang, 2013; An et al., 2019). Stainless steel guide cannulae (22 gauge; Plastics One, Inc.) were bilaterally implanted to the dorsal HPC [anterior–posterior (AP), −3.3 mm; mediolateral (ML), ± 2.2 mm; dorsoventral (DV), 2.4–2.8 mm from the dorsal surface of the brain]. Obdurators (30-gauge, Plastics One, Inc.) were inserted into the guide cannula to prevent obstruction. Rats were treated with Anafen immediately following surgery and allowed to recover for at least 7 days.

Infusions were performed by inserting custom needles (30-gauge, Small Parts, Inc.) connected through a PE-50 tube into an infusion pump (Harvard Apparatus), extended 1.0 mm past the end of the cannulae. Acetylcholine chloride or ACSF was infused into the HPC (0.5 μl/min per side for 2 min) 30 min before testing began. The needles were left for 3–5 min to allow the diffusion of the drug. One week before the treatment, the infusion procedure was habituated on four separate days. The infusion sites were identified with the aid of The Rat Brain in Stereotaxic Coordinates (1997, third edition).

### LFP Recording

Microelectrodes were arranged in two 4 × 4 matrices using a 25-μm diameter platinum/iridium wire coated with polyimide (California Fine Wire Company) in a 16-gauge silica tube (World Precision Instruments). It was then attached *via* gold pins to an EIB-36-PTB board (Neuralynx, Inc.), which was assembled to a microdrive (Harlan 8-drive; Neuralynx). The electrode tips were gold-plated to maintain the impedance to 200–600 kΩ measured at 1 kHz (NanoZ, Neuralynx).

Rats were anesthetized with isoflurane and prepared for surgery using previously reported procedures (An et al., 2013a; Sun et al., 2018a, 2019). Two electrode arrays were chronically implanted: one located at the CA1 region (AP, -3.5 mm; ML, 2.5 mm; DV, 2.0 mm) and the other one was located at the CA3 region (AP, -4.2 mm; ML, 3.5 mm; DV, 2.5 mm) of the HPC. The left or right hemisphere was implanted randomly, but counterbalanced across groups.

The recording was conducted with a Digital Cheetah system (Cheetah Software, Neuralynx, Inc.), sampled at 32 kHz, and filtered at 0.1–9,000 Hz. The animals’ behavior was monitored by a digital ceiling camera (Neuralynx, Inc.) and the CCD camera’s signal was fed to a frame grabber (sampling rate, 64 Hz) with the experimental time superimposed for off-line analysis. Fast movements, which were defined as any movement with a velocity higher than 5 cm/s, were scored using the EthoVision XT software (Noldus Information Technology). LFPs were recorded during the whole behavioral tests. Since the hippocampal LFP is highly sensitive to the behavioral state, the signal from the behavioral test and home cage environment was analyzed only when the rats’ velocity was higher than 5 cm/s. Before analysis, directed coherence (DC) offsets and slow fluctuations were eliminated by applying the *locdetrend* function in the Chronux 2.00 toolbox (Bokil et al., 2010), which subtracts the linear regression line fit with the following parameters: 1-s window size, 50-ms time step, time–bandwidth product of 5, and a taper count of 9. The Butterworth band-pass filter was used for all bands of interest, including delta (0.5–3 Hz), low theta (4–7 Hz), high theta (8–12 Hz), beta (14–30 Hz), and gamma (52–120 Hz). The power spectral densities were calculated using a fast Fourier transform-based Welch’s method (1,024 frequencies between 1 and 200 Hz, smoothed with a Gaussian kernel with bin width of 3). The analyses were performed with Neuroexplorer, Matlab (MathWorks) software.

After the completion of all recording sessions, the electrode and cannula sites of each rat were marked by electrolytic lesions (10 μA current for 10 s). Rats were sacrificed by urethane overdose and perfused transcardially with saline followed by 10% formalin. The brains were removed and post-fixed in a 10% formalin 10% sucrose solution. Brains were sectioned and the recording sites were identified using standard protocols with reference to The Rat Brain in Stereotaxic Coordinates (1997, third edition). Only data from rats with probes contained within the hippocampus were collected (Figure 1).

### Phase Locked Value

Phase locked value (PLV) is defined to analyze the strength of phase synchronization. Extracting the phase of two signals, ϕ_*a*_ and ϕ_*b*_ were obtained. PLV is defined as follows:

$$P\,L\,V=\left|\frac{1}{N}\,\sum_{j=1}^{N}\exp\left(i\,\left[\phi_{a}\,\left(j\,\Delta \,t\right)-\phi_{b}\,\left(j\,\Delta \,t\right)\right]\right)\right|$$

where *N* stands for the length of the time series and $\frac{1}{\Delta \,t}$ is the sampling frequency. The value of PLV is between 0 and 1, meaning that 1 indicates full synchronization and 0 no syncing at all.

### gPDC Algorithm

PDC, whose defination is based on the notion of a linear Granger causality, is proposed to describe the causal relationship between multivariate time series. Its core meaning is based on the decomposition of multivariate partial coherences computed from multivariate autoregressive models. A two-variate process PDC algorithm was introduced in the following.

Considering a two-dimensional process,

(1) $$X\left(t\right):=\left[x_{1}\left(t\right)\_\;\_\_x_{2}\left(t\right)\right]$$

Granger causality within a two-variate process defined by *X*(*t*) is assessed by modeling them through a vector autoregressive (VAR) model of the form:

(2) $$X\,\left(\text{t}\right)=\sum_{r=1}^{p}A_{r}\,X\,\left(t-r\right)+E\,\left(t\right)$$

with

(3) $$A_{r}=\left[\begin{array}{cc} a_{11}\,\left(r\right) & a_{12}\,\left(r\right)\\ a_{21}\,\left(r\right) & a_{22}\,\left(r\right) \end{array}\right]$$

Taking the Fourier transformation of the VAR coefficients:

$$A\,\left(f\right)=\sum_{r=1}^{p}A_{r}\cdot \exp\left(-i\,2\,\pi \,f\,r\right)$$

yields a frequency domain representation of the VAR model.

Defining the matrix: A-(f)=I-∑r=1pAr⋅exp⁡(-i⁢2⁢π⁢f⁢r)=[a1-1(f) a2-2(f)]as the difference between the identity matrix.

And then PDC from variables **x*_*j*_* to *x*_*i*_ is defined as:

(4) πi⁢j⁢(f)=ai⁢j-(f)a-(f)jHaj-(f)

It has been shown that large differences in the variances of the modeled time series can lead to distortions in the resulting PDC values (Winterhalder et al., 2005; Baccala et al., 2007). To avoid this, a variation of the original PDC, which is called *generalized PDC* (gPDC) (Baccala et al., 2007), is presented. In gPDC, the coefficients $\overset{-}{\underset{i\,j}{A}}\left(f\right)$ are normalized by the standard deviation of the *E*(*t*) model residuals:

(5) πi⁢jg⁢(f)=1σia-(f)i⁢j1σ12A-(f)1⁢jA-(f)H1⁢j+1σ22A-(f)2⁢jA-(f)H2⁢j

The denominator in Equation (5) is a normalization that bounds the gPDC coefficients to values from 0 to 1. The choice of scaling means that $\left|\pi_{i\,j}^{\text{g}}\,\left(f\right)\right|$ measures the outflow of information from signal *x*_*j*_ to signal*x*_*i*_, with respect to the total outflow of information from *x*_*j*_ to all signals.

### Data and Statistical Analysis

All the data were expressed as the mean ± SEM. Two- or one-way ANOVA was applied for the analysis of PLV, gPDC, and the behavioral test, followed by Tukey’s *post hoc* test. An independent samples *t* test was used to analyze the ACh and AChE levels. The analyses were performed using SPSS 16.0 software and the significant level was set at 0.05.

## Results

### ACh and AChE Levels in Melamine-Treated Rats

**FIGURE 2:**
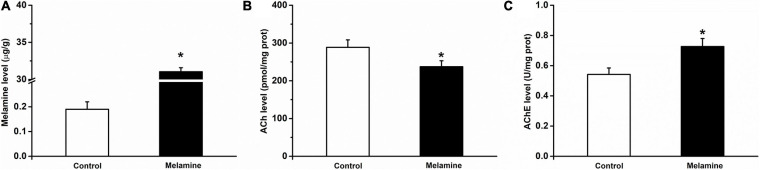
Melamine, ACh and AChE levels in the HPC of melamine-treated rats. **(A)** The melamine level was significantly enhanced in the HPC of melamine-treated rats. Following the Y-maze test, **(B)** the expression of ACh was reduced and **(C)** the activity of AChE was enhanced in the HPC. *P < 0.05, melamine vs. control.

Compared to vehicle treatment of the control group, a high concentration of melamine was found in the melamine group, which indicated that melamine could gather in the HPC (*t*_14_ = 30.8, 31.05 ± 0.53 vs. 0.19 ± 0.03 μg/g, *P* = 0.000; Figure 2A). After spatial learning, the ACh and AChE activities were assessed. It can be seen that the ACh level (*t*_14_ = 2.9, 288.60 ± 19.61 vs. 237.40 ± 15.73 pmol/mg, *P* = 0.012; Figure 2B) in the melamine group was significantly reduced, while the AChE activity (*t*_14_ = 4.9, 0.55 ± 0.04 vs. 0.72 ± 0.05 U/mg, *P* = 0.000; Figure 2C) was significantly increased compared with those of the control group.

### The Strength of Phase Synchronization

**FIGURE 3:**
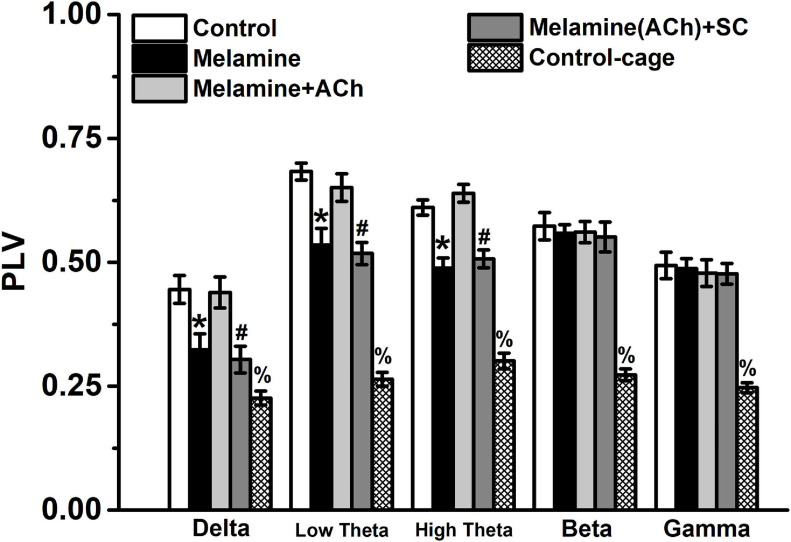
The phase locked value (PLV) between the CA3 and CA1 regions. The PLV was significantly declined comparing melamine to the control group. Injection of acetylcholine (ACh) into the hippocampus (HPC) effectively enhanced the PLV index. *P < 0.05, melamine vs. control, melamine+ACh, and control cage; ^#^*P* < 0.05, melamine(ACh)+SC vs. control, melamine+ACh, and control cage; ^%^*P* < 0.05, control cage vs. other groups.

Compared with the control group, the values of PLV at the low-frequency bands, including delta, low theta, and high theta, were significantly reduced following chronic melamine exposure [two-way ANOVA, interaction effect between treatment and band: *F*_(__16_,_60__)_ = 12.27, *P* < 0.001; *post hoc*: control vs. melamine, delta 0.445 ± 0.0283 vs. 0.324 ± 0.032, low theta 0.683 ± 0.017 vs. 0.535 ± 0.034, high theta 0.611 ± 0.016 vs. 0.488 ± 0.020, all *P* < 0.05] (Figure 3). Comparing melamine+ACh with the melamine groups, the infusion of ACh could effectively enhace the declined PLV (melamine+ACh vs. melamine, delta 0.439 ± 0.031 vs. 0.324 ± 0.032, low theta 0.651 ± 0.028 vs. 0.535 ± 0.034, high theta 0.639 ± 0.018 vs. 0.488 ± 0.020, all *P* < 0.05), while comparing melamine(ACh)+SC with the melamine+ACh groups, scopolamine, an acetylcholine receptor antagonist, markedly reversed the ACh effects [melamine+ACh vs. melamine(ACh)+SC: delta, 0.439 ± 0.031 vs. 0.304 ± 0.027; low theta, 0.651 ± 0.028 vs. 0.518 ± 0.022; high theta, 0.630 ± 0.018 vs. 0.507 ± 0.018; all *P* < 0.05].

**FIGURE 4:**
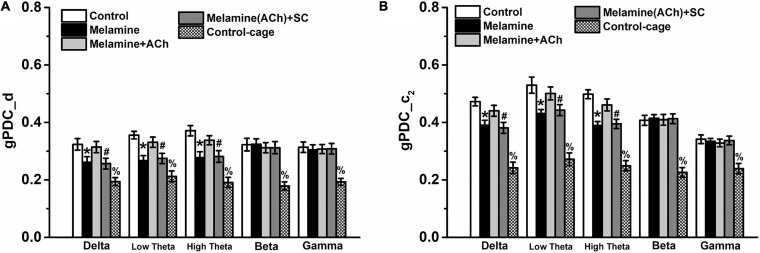
Directional coupling index d and c_2_ in the CA3–CA1 pathway. **(A)** Coupling direction index d between CA1 and CA3. CA3 predominantly drives CA1 in the case of *d* > 0. **(B)** Unidirectional influence index c*_2_* indicating unidirectional neural information flow (NIF) from CA3 to the CA1 area. Both indexes *d* and *c*_2_ were decreased, but could be reversed by intrahippocampal infusion of acetylcholine (ACh). *P < 0.05, melamine vs. control, melamine+ACh, and control cage; ^#^*P* < 0.05, melamine(ACh)+SC vs. control, melamine+ACh, and control cage; ^%^*P* < 0.05, control cage vs. other groups.

In the analysis of gPDC in the CA3–CA1 pathway, the directionality index *d* of NIF in the melamine group was markedly diminished compared with that of the control group at the delta, low theta, and high theta frequency bands [two-way ANOVA, interaction effect between treatment and band: *F*_(__16_,_60__)_ = 10.22, *P* < 0.001; *post hoc*: control vs. melamine, delta 0.324 ± 0.020 vs. 0.261 ± 0.019, low theta 0.356 ± 0.013 vs. 0.267 ± 0.018, high theta 0.371 ± 0.018 vs. 0.278 ± 0.020, all *P* < 0.05] (Figure 4A). Meanwhile, the values of the unidirectional influence*c*_2_, indicating the unidirectional coupling from CA3 to CA1, were significantly decreased comparing melamine with the control groups [two-way ANOVA, interaction effect between treatment and band: *F*_(__16_,_60__)_ = 10.98, *P* < 0.001; *post hoc*: control vs. melamine, delta 0.472 ± 0.015 vs. 0.390 ± 0.017, low theta 0.530 ± 0.028 vs. 0.431 ± 0.013, high theta 0.498 ± 0.016 vs. 0.390 ± 0.014, all *P* < 0.05] (Figure 4B). Comparing melamine+ACh with the melamine groups, the injection of ACh into the hippocampus could mitigate the melamine-induced weakened NIF, indicated by the enhancement in the directinonal *d* (melamine+ACh vs. melamine: delta, 0.314 ± 0.019 vs. 0.261 ± 0.019; low theta, 0.331 ± 0.018 vs. 0.267 ± 0.018; high theta, 0.337 ± 0.016 vs. 0.278 ± 0.020; all *P* < 0.05) and the unidirectinal *c*_2_ index of NIF (melamine+ACh vs. melamine: delta, 0.440 ± 0.019 vs. 0.390 ± 0.017; low theta, 0.501 ± 0.022 vs. 0.431 ± 0.013; high theta, 0.461 ± 0.021 vs. 0.390 ± 0.014; all *P* < 0.05). Furthermore, comparing melamine(ACh)+SC with the melamine+ACh groups, scopolamine could effectively block the reversal effects of ACh on the directinonal *d* [melamine+ACh vs. melamine(ACh)+SC: delta, 0.314 ± 0.019 vs. 0.256 ± 0.019; low theta, 0.331 ± 0.018 vs. 0.274 ± 0.018; high theta, 0.338 ± 0.016 vs. 0.282 ± 0.020; all *P* < 0.05] and the unidirectinal *c*_2_ index of NIF [melamine+ACh vs. melamine(ACh)+SC: delta, 0.440 ± 0.019 vs. 0.382 ± 0.019; low theta, 0.501 ± 0.022 vs. 0.446 ± 0.019; high theta, 0.461 ± 0.021 vs. 0.397 ± 0.016; all *P* < 0.05].

Additoinally, the values of PLV, the directionality index *d*, and the unidirectinal *c*_2_ index of control cage rats were significantly lower than those of the control (PLV: control vs. control cage, delta 0.445 ± 0.028 vs. 0.226 ± 0.014, low theta 0.683 ± 0.017 vs. 0.264 ± 0.014, high theta 0.611 ± 0.016 vs. 0.301 ± 0.016, beta 0.573 ± 0.028 vs. 0.273 ± 0.012, gamma 0.494 ± 0.027 vs. 0.247 ± 0.010, all *P* < 0.05; gPDC-d: control vs. control cage, delta 0.324 ± 0.020 vs. 0.194 ± 0.014, low theta 0.356 ± 0.013 vs. 0.212 ± 0.019, high theta 0.371 ± 0.017 vs. 0.191 ± 0.0018, beta 0.322 ± 0.022 vs. 0.179 ± 0.014, gamma 0.314 ± 0.018 vs. 0.193 ± 0.012, all *P* < 0.05; gPDC-c_2_: control vs. control cage, delta 0.472 ± 0.015 vs. 0.242 ± 0.008, low theta 0.540 ± 0.028 vs. 0.272 ± 0.011, high theta 0.498 ± 0.016 vs. 0.249 ± 0.008, beta 0.407 ± 0.017 vs. 0.226 ± 0.013, gamma 0.342 ± 0.015 vs. 0.239 ± 0.009, all *P* < 0.05) and melamine-treated rats (PLV: melamine vs. control cage, delta 0.324 ± 0.032 vs. 0.226 ± 0.014, low theta 0.535 ± 0.034 vs. 0.264 ± 0.014, high theta 0.488 ± 0.020 vs. 0.301 ± 0.016, beta 0.559 ± 0.018 vs. 0.273 ± 0.012, gamma 0.488 ± 0.020 vs. 0.247 ± 0.010, all *P* < 0.05; gPDC-d: melamine vs. control cage, delta 0.261 ± 0.020 vs. 0.194 ± 0.014, low theta 0.267 ± 0.018 vs. 0.212 ± 0.019, high theta 0.278 ± 0.020 vs. 0.191 ± 0.0018, beta 0.324 ± 0.019 vs. 0.179 ± 0.014, gamma 0.305 ± 0.017 vs. 0.193 ± 0.012, all *P* < 0.05; gPDC-c_2_: melamine vs. control cage, delta 0.390 ± 0.017 vs. 0.242 ± 0.008, low theta 0.431 ± 0.013 vs. 0.272 ± 0.011, high theta 0.389 ± 0.014 vs. 0.249 ± 0.008, beta 0.415 ± 0.012 vs. 0.226 ± 0.013, gamma 0.334 ± 0.010 vs. 0.239 ± 0.009, all *P* < 0.05), indicating that all the indexes were spatial task-related.

Our findings support melamine exposure declining the NIF from CA3 to CA1 in the hippocampus, and ACh activity plays a central role in the synaptic function.

### Spatial Learning in the Y-Maze Test

**FIGURE 5:**
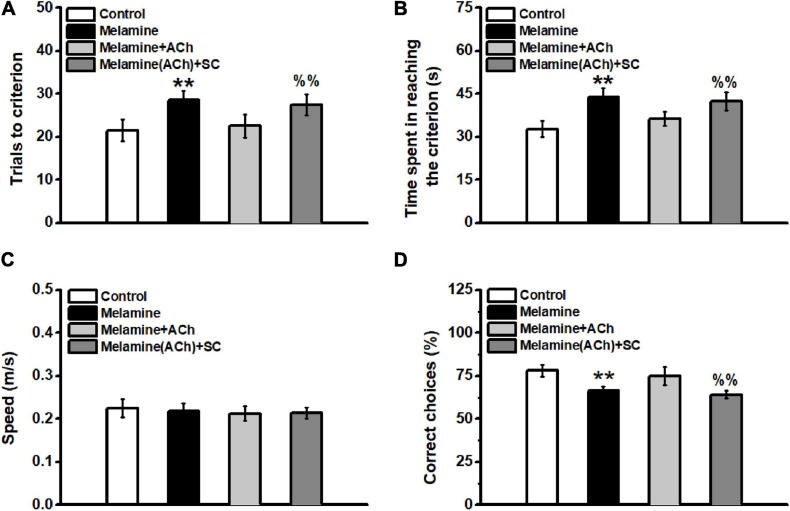
Spatial learning in the Y-maze task. **(A)** Total trials to the criterion. The total trails to the criterion of the melamine group were significantly increased compared to the control. Injection of acetylcholine (ACh) mitigated the spatial learning deficits by reducing the total trial numbers during spatial training. **(B)** Total time spent in reaching the criterion. No statistical difference was found among all groups. **(C)** Velocity of animals during the whole behavioral test. No difference was found among groups. **(D)** Percentage of correct trials during the memory test. **P < 0.01, melamine vs. control and melamine+ACh; ^%%^*P* < 0.01, melamine(ACh)+SC vs. control and melamine+ACh.

In the spatial training, it can be seen that melamine exposure significantly increased the total trials to the criterion [one-way ANOVA, effect of treatment: *F*_(__3_,_25__)_ = 26.56, *P* < 0.001; *post hoc*: control vs. melamine, 21.5 ± 2.6 vs. 28.5 ± 2.2, *P* < 0.01] (Figure 5A), while ACh treatment rescued the impairment of learning ability (melamine+ACh vs. melamine, 22.5 ± 2.7 vs. 28.5 ± 2.2, *P* < 0.01). Compared with the melamine+ACh group, the number of trials to the criterion of melamine(ACh)+SC group was significantly higher [melamine+ACh vs. melamine(ACh)+SC, 22.5 ± 2.7 vs. 27.5 ± 2.4, *P* < 0.01]. Similarly, the total time spent in reaching the criterion was significantly enhanced comparing melamine to the control groups [one-way ANOVA, effect of treatment: *F*_(__3_,_25__)_ = 33.85, *P* < 0.001; *post hoc*: control vs. melamine, 32.7 ± 2.7 vs. 43.9 ± 3.0, *P* < 0.01] (Figure 5B), while ACh treatment could effectively reduce the learning time of melamine rats (melamine+ACh vs. melamine, 36.3 ± 2.4 vs. 43.9 ± 3.0, *P* < 0.01). The total time of the melamine(ACh)+SC group was statistically higher than that of the melamine+ACh group [melamine(ACh)+SC vs. melamine+ACh, 42.4 ± 3.1 vs. 36.3 ± 2.4, *P* < 0.01]. However, the moving speeds among groups remained constant throughout testing, with no statistical difference found [one-way ANOVA, effect of treatment: *F*_(__3_,_25__)_ = 0.47; control, 0.226 ± 0.021 m/s; melamine, 0.218 ± 0.018 m/s; melamine+ACh, 0.212 ± 0.017 m/s; melamine(ACh)+SC, 0.213 ± 0.013 m/s; *P* > 0.05] (Figure 5C).

Additionally, the spatial memory of the subgroups of rats was tested 1 week following the training day. There was no statistical difference between melamine+ACh and the control groups [one-way ANOVA, effect of treatment: *F*_(__3_,_13__)_ = 18.56, *P* < 0.001; *post hoc*: control vs. melamine+ACh, 78.0 ± 3.4 vs. 75.0 ± 5.3, *P* > 0.05] (Figure 5D). Meanwhile, the percentage of correct choice of the melamine group was significantly lower than those of the control and melamine+ACh groups (melamine vs. control, 63.3 ± 2.4 vs. 78.0 ± 3.4, *P* < 0.01; melamine vs. melamine+ACh, 63.3 ± 2.4 vs. 75.0 ± 5.3, *P* < 0.01). Furthermore, scopolamine treatment significantly decreased the percentage of the correct trials of melamine+ACh-treated rats [melamine+ACh vs. melamine(ACh)+SC, 75.0 ± 5.3 vs. 64.0 ± 2.2, *P* < 0.01].

## Discussion

The 4-week intragastric administration induces the accumulation of melamine in the HPC. Meanwhile, chronic melamine exposure decreases the activities of ACh and its esterolytic enzyme in the HPC. The enhanced melamine disrupts neural coupling and reduces the NIF from the CA3–CA1 pathway, which is involved in spatial learning impairment. Furthermore, intrahippocampal injection of ACh could enhance the decreased directional and unidirectional coupling indexes, thereby rescuing the learning deficits. Furthermore, the infusion of scopolamine, an acetylcholine receptor antagonist, effectively blocked the mitigative effects of ACh treatment. Our findings identify for the first time a specific neurotransmitter system involved in melamine-induced neural coupling dysfunction.

Previous evidence indicate that melamine can pass the BBB and enter the brain (Wu et al., 2009). However, a low dose of melamine (5 mg/kg) had no effect on locomotor activity in the open-field test in both young and adult animals (Yang et al., 2012). Chronic medium dose of melamine (300 mg/kg) could significantly elevate the melamine content in the HPC to 32.82 ± 0.80 μg/g and increase the incidences of neurological and behavioral deficits (An et al., 2012a). Consistent with our previous findings, melamine can accumulate in the brain, which is indicated by the abnormal melamine concentrations found in the hippocampus of the melamine-treated group (An et al., 2011, 2015; An and Sun, 2017a).

Although the time spent in reaching the criterion is prolonged by melamine treatment, melamine-treated rats can effectively achieve the learning. HPC activity is necessary to correctly perform spatial navigation tasks (Kleinknecht et al., 2012; Zhang and Manahan-Vaughan, 2015). Our results confirm and expand these findings by demonstrating that HPC integrity is necessary for the acquisition of spatial behavior. Indeed, HPC lesions increase the percentage of, mainly procedural, errors during training (Moussa et al., 2011). Accordingly, melamine-treated rats are less efficient in acquisition. However, although procedural performance was consistently impaired throughout all training sessions, previous studies have reported that melamine-treated rats could learn in the water maze task after sufficient training (An and Sun, 2017a, 2018). Further research should investigate whether working memory is impaired following chronic melamine treatment.

Synchronized activities in different frequency bands are involved in regulating the flow of neural information during cognitive processes (Salinas and Sejnowski, 2001; Gallinat et al., 2006). During spatial exploratory behavior and increased levels of acetylcholine, both low theta and high theta oscillations are particularly prominent in the hippocampus (Benchenane et al., 2010; Aitken et al., 2017). Specifically, optimal conditions have been proposed to be created by the theta rhythm to enhance synchrony among neural networks, leading to synaptic changes necessary for memory formation (Douchamps et al., 2013). This was consistent with data showing that, at a general level, the findings are consistent both with views applying the modulation of memory formation to a systems-level understanding of memory processing (Chang and Gold, 2003) and with views considering ACh to be a regulator of attention (Everitt and Robbins, 1997). Previously, typical, highly regular theta activity with occasional intermittent delta activity periods was found in behavior of the walking state (Keita et al., 2000). Furthermore, hippocampal cholinergic activation is of importance for amplitude modulation of the theta and delta components of hippocampal LFP (Keita et al., 2000). The amplitude of delta response was obviously enhanced during the oddball task, indicating that delta oscillation is related to signal detection and decision making (Basar et al., 2001). Facilitation of coherent oscillations in the delta frequency band could promote the induction of short-term plasticity (Kiss et al., 2011), which can increase or decrease the synaptic efficacy and memory consolidation. Confirming previous findings, the higher level of ACh in the HPC is consistent with the enhancement of the neural activity of HPC cholinergic cells (Benardo and Prince, 1982; Teles-Grilo Ruivo and Mellor, 2013), and the neural correlate is associated with variations in the oscillatory activity during the phenotype of the spatial test (Hasselmo, 2006; Picciotto et al., 2012).

Acetylcholine in the hippocampus, which plays a central role in learning and memory functions, reduced drastically in the melamine group. By rapidly hydrolyzing ACh in the cholinergic system, AChE functions to terminate synaptic transmission. Our findings are in agreement with the findings that the improvement in memory function can be found after enhancing the cholinergic tone with the cholinesterase inhibitor, which reduces acetylcholine breakdown (Mans et al., 2014; Gawel et al., 2016; Zueva et al., 2019). On the other hand, pharmacological impairments of cholinergic transmission disturb theta synchrony, implying that cholinergic projections in the HPC are the impartible region during theta generation (Goutagny et al., 2009). In concordance with our ethological results, recent findings reported that ACh was associated with theta oscillations to promote neural function such as learning and memory (Sparks and Chapman, 2013; Damodaran et al., 2019). Therefore, combined theta oscillations along with neurotransmitter transmissions and all those aforementioned changes indicate the tight correlation with theta coupling in the CA3–CA1 pathway. It suggested that the theta rhythm activity might mediate neuronal coupling between the CA3 and the CA1 region *via* cholinergic systems; hence, based on this frequency band, the directional index could be used as a novel representation of synaptic plasticity in the CA3–CA1 pathway, manifesting the cognitive and memorial functional alterations. Together, our findings may imply that melamine-induced spatial cognitive impairments are associated with reductions in hippocampal ACh expression. Nevertheless, this needs to be verified in other memory tests or a more complex hippocampus-dependent task since memory tasks evaluated in a single day may be not enough.

Recently, high-resolution measurements of ACh release indicate that the release may be precisely timed (Parikh et al., 2007; Howe et al., 2013), leading to an efficient neural information flow by optimizing spike time-dependent plasticity (Edelmann and Lessmann, 2013). Indeed, to activate its receptors, ACh exerts its function in neural excitability/inhibitory balance and neurotransmission (Teles-Grilo Ruivo and Mellor, 2013). Consistently, neural correlates regulate synaptic function through spike timing-dependent mechanisms and enhancing the memory of the attended stimuli (Hasselmo, 2006; Picciotto et al., 2012). Both *ex vivo* and *in vivo* findings provided evidence that low frequencies such as low theta and high theta bands can efficiently facilitate glutamatergic inputs into neuronal dendrites and enhance dendritic invasion, leading to the induction and maintenance of long-term plasticity (Selbach et al., 2004; Vandecasteele et al., 2014). We have also found that both acute and chronic melamine exposure could inhibit acetylcholinergic transmission and disturb the theta burst-induced synaptic excitability in the hippocampal CA1 area (An and Sun, 2017c, 2018). Therefore, melamine may impair the impacts of ACh on hippocampal networks and its stable oscillatory, which can effectively facilitate information processing and memory consolidation. However, more evidence is needed to support this hypothesis.

In summary, chronic melamine exposure impairs HPC-dependent spatial learning and disrupts the expression of ACh and AChE in the HPC. Meanwhile, the coupling directional index based on LFP was reduced robustly in low-frequency bands, including delta, low theta, and high theta oscillations. The unidirectional index *c*_2_ showed that CA3 driving CA1 was critically decreased, indicating that the cognitive dysfunction could be in part caused by the reduction of NIF in the hippocampal CA3–CA1 pathway. Furthermore, the injection of ACh into the HPC could reverse the weakened NIF and cognitive deficits of the melamine-treated rats, indicating that the reduction in ACh expression is attributed to the impairments of neural and cognitive dysfunction. Meanwhile, scopolamine treatment could completely block these mitigative effects. Our findings, for the first time, support the cellular mechanisms underlying the impairments of neural oscillation and coupling by melamine exposure.

## Data Availability Statement

The data that support the findings of this study are available in the Supplementary Material and further data can be reached from the corresponding author upon reasonable request.

## Ethics Statement

The animal study was reviewed and approved by the Ethics Committee on the Care and Use of Animals Committee of Guizhou University of Traditional Chinese Medicine.

## Author Contributions

WS and PL conducted the experiments. CT and LA conceived and designed the experiments and guided the research direction. WS, PL, and LA wrote the manuscript. All authors contributed to the article and approved the submitted version.

## Conflict of Interest

The authors declare that the research was conducted in the absence of any commercial or financial relationships that could be construed as a potential conflict of interest.
