# Supplementary material for: Melamine Disrupts Acetylcholine-Mediated Neural Information Flow in the Hippocampal CA3–CA1 Pathway
Source: Front Behav Neurosci. 2021 Feb 18;15:594907. doi: 10.3389/fnbeh.2021.594907 (PMC7930216; doi:10.3389/fnbeh.2021.594907)
Supplement: Supplementary file 1 [file Data_Sheet_1.pdf]

Supplementary information

SI Results.

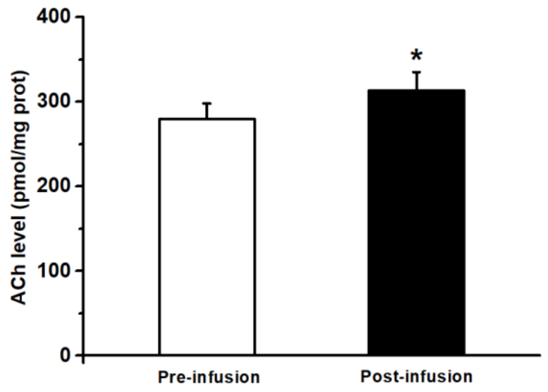

Fig. S1. Thirty min after intrahippocampal infusions of acetylcholine (5.0 nM, 0.25 μL/side), the acetylcholine level was significantly increased (t_6=2.7, P=0.036).
